# Cancer Research

**Published:** 1904-01-30

**Authors:** 


The Hospital.

A Journal of

The Medical Sciences and Hospital Administration.

Vol. XXXV.—No. 905. JANUARY 30, 1904.

### Cancer Research.

We fear there may be just a suggestion of prematurity about the communications on the subject of Cancer Research which last week found their way into the columns of the non-medical press. Certain papers were communicated to the Royal Society, and, we believe, were not read or discussed, but only, on account of the pressure of other business, placed before the Fellows in printed form. One of them, by Dr. E. F. Bashford and Mr. J. A. Murray, was entitled "The Significance of the Zoological Distribution, the Nature of the Mitoses, and the Transmissibility of Cancer," and represented some of the results of the work done by the Cancer Research Fund during the past year; while the other, by Professor Farmer, Mr. Moore, and Mr. Walker, was an independent communication in continuation of an earlier one upon the same subject, and was entitled "Resemblances Exhibited Between the Cells of Malignant Growths in Man and those of Normal Reproductive Tissue." We presume that, as far as the readers of the great daily papers are concerned, "Mitoses" might just as well be "Mesopotamia." Many of these readers would know, and all could easily find out, that μίτος signifies a thread, but we greatly question whether this knowledge would bring them any nearer to the fact that the derived word is used, in the jargon of biology, to signify the splitting of the nuclear chromatin of cells undergoing indirect division, or that they would learn anything more, from the published summary of the paper, than that there seems to be some prospect of establishing a definite relation between cancerous growths and some of the processes of ordinary nutrition, when these are interfered with by certain external conditions. The authors of the second paper describe influences under which the prothallia of certain ferns retain their characteristics instead of passing on into the ordinary sexual development; and the consequent growth of prothallium, adhering to a frond, is in many respects similar to the growth of malignant tumours in the animal body. An additional fact of importance seems to be that the cancer of one species cannot be transmitted to another, while it can often be transmitted to animals of the same species; and hence it would appear that the essential character of the disease must be a perversion of the ordinary cell growth of the affected body, only to be reproduced in cells of the same character as those in which it originated.

All this is eminently satisfactory as far as it goes, and might with great propriety have been placed before the readers of a medical journal, who would not expect too much from it, and who would know that there is often a long interval between scientific hypotheses and the fruition ultimately to be derived from them. "What is the use," would say the average Philistine, "of talking to me about the 'Mitoses' of cancer? Have you got a pill that will cure it? and if not, in the words of an old legal formula, why not, and how otherwise?" Faraday somewhere says that the world little knows how many of the thoughts and theories which have passed through the mind of a scientific investigator have been crushed in silence and secrecy by his own severe criticism and adverse examination, and that in the most successful instances not a tenth of the suggestions, the hopes, the wishes, the preliminary conclusions have been realised. On such grounds as these, while we rejoice to receive evidence that investigation concerning cancer is being actively pursued, we greatly question the desirability of bringing the successive steps of the process under the notice of the readers of newspapers, who, as a rule, will be absolutely incompetent to understand their nature or to appreciate their value. At the present stage of the inquiry, it is surely sufficient to say that it is being pursued with care and diligence, and that it may be taken as nearly established that the disease is not due to any invasion from without, to any parasite or bacterium, but solely to a perversion of the ordinary course of nutrition in the body of the subject. We do not see that the views advanced by the authors now referred to are so entirely antagonistic to those of Cohnheim as those authors appear to suppose; because, if there be anything in animal embryology corresponding to the prothallium stage of a fern or an equisetum, the undue persistence of this stage, even to the advanced period of life at which cancer usually commences, seems to point in the direction of the dormancy of embryonic tissue, and of its sudden springing into unnatural activity. If the analogy between the persistent prothallium and cancer be ultimately established, much will still have to be done in the direction or research into the conditions by which the abnormality is occasioned and sustained, and of the methods by which the occurrence of these conditions may be hindered or prevented. In the way of any direct remedy, other than that of early removal, the tendency of modern work is not encouraging; and, so far, at least, there seems no reason to suppose that either radium, or any of the new "rays" which are constantly being provided by science, will be more useful than the surgeon's knife in cases in which the size and situation of the growth will permit the latter to be applied. We have nothing but praise for the efforts which are being made in these directions; but, until they are more advanced than at present seems to be the case, we should be glad to see them excluded from any but professional journals.

